# Dissociated Humoral and Cellular Immune Responses to Recombinant Zoster Vaccine in Myeloproliferative Neoplasms Under JAK Inhibition: A Pilot Study

**DOI:** 10.3390/ijms27125543

**Published:** 2026-06-19

**Authors:** Julio Torres-González, Blanca O’Donnell-Cortés, Rodolfo Matías Ortiz-Flores, Dariusz Piotr Narankiewicz Talarczyk, Borja Cidoncha-Morcillo, Fernando Fariñas-Guerrero, María Rodríguez-González, Regina García-Delgado, Alejandro Escamilla-Sánchez

**Affiliations:** 1 BE21-Hematología e Inmunoterapia, IBIMA Plataforma BIONAND, Avenida Severo Ochoa 35, 29590 Málaga, Spain; julio.torres@ibima.eu (J.T.-G.); regina.garcia.delgado.sspa@juntadeandalucia.es (R.G.-D.); jandromilla@uma.es (A.E.-S.); 2 Departamento de Salud Pública y Psiquiatría, Universidad de Málaga, 29010 Málaga, Spain; blanca.odonnell.sspa@juntadeandalucia.es; 3 Área A-23 Medicina Preventiva y Salud Pública, IBIMA Plataforma BIONAND, Instituto de Investigación Biomédica de Málaga, Avenida Severo Ochoa 35, 29590 Málaga, Spain; dariusz.narankiewicz.sspa@juntadeandalucia.es; 4 UGC Hematología y Hemoterapia, Hospital Universitario Virgen de la Victoria, Campus de Teatinos, s/n, 29010 Málaga, Spain; borcm@hotmail.es (B.C.-M.); mariarodriguezg92@gmail.com (M.R.-G.); 5 Instituto de Hematología, Inmunología y Biología Médica Avanzada (HEMOINMUN), 29590 Málaga, Spain; farinas.infecciosas@gmail.com; 6 Departamento de Fisiología Humana, Histología Humana, Anatomía Patológica y Educación Físico-Deportiva, Facultad de Medicina, Universidad de Málaga, Boulevard Louis Pasteur 32, 29010 Málaga, Spain

**Keywords:** myeloproliferative neoplasms, recombinant zoster vaccine, JAK inhibitors, humoral immunity, cellular immunity, herpes zoster

## Abstract

Patients with myeloproliferative neoplasms (MPN) are at increased risk of herpes zoster, particularly during Janus kinase inhibitor (JAKi) therapy, yet the immunogenicity of recombinant zoster vaccine (RZV) in this setting remains incompletely characterized. We performed a prospective pilot translational study including 18 patients with MPN and a small age-matched healthy donor group (*n* = 4, descriptive reference only). Samples were collected at baseline, 21 days after the first dose, and 21 days after the second dose. Humoral response was assessed by anti-varicella-zoster virus (VZV) immunoglobulin G (IgG) enzyme-linked immunosorbent assay (ELISA), whereas antigen-specific cellular responses were evaluated after ex vivo stimulation with recombinant VZV glycoprotein E followed by flow cytometry and cytokine quantification. IgG levels increased over time in MPN patients, while cellular responses remained limited, heterogeneous, and not consistently enhanced. Cytokine production was low and variable across time points. Overall, RZV in MPN under JAKi was associated with detectable humoral responses but limited cellular activation, supporting an apparent discordance between humoral and cellular immune readouts under the experimental conditions used.

## 1. Introduction

Herpes zoster (HZ) is a viral infection caused by the reactivation of varicella-zoster virus (VZV), which remains latent in the sensory nerve ganglia following primary infection. Reactivation leads to a painful, unilateral vesicular rash distributed along the affected dermatomes and represents a significant clinical burden, particularly in older adults [1,2,3,4]. The incidence of HZ increases markedly with age, largely due to the progressive decline in VZV-specific T-cell-mediated immunity [4,5,6].

Patients with myeloproliferative neoplasms (MPNs), across disease subtypes, have an increased risk of HZ [7]. These disorders are characterized by clonal haematopoiesis and aberrant activation of the Janus kinase/signal transducer and activator of transcription (JAK/STAT) pathway, which plays a central role in hematopoietic regulation and immune function [8,9,10]. Treatment with JAK inhibitors (JAKi), such as ruxolitinib, fedratinib or momelotinib, further impairs immune competence in addition to disease-related immune dysregulation. These agents affect both innate and adaptive immune responses, including T-cell and natural killer (NK) cell function, thereby increasing susceptibility to viral infections, including VZV reactivation [11,12,13,14,15,16].

Given this increased vulnerability, vaccination against VZV represents a key preventive strategy in patients with MPN. The recombinant zoster vaccine (RZV, Shingrix^®^), which is based on the VZV glycoprotein E (gE) antigen combined with the AS01B adjuvant system, has emerged as a suitable option for immunocompromised individuals because it does not contain live virus [17,18]. The AS01B adjuvant promotes the activation of antigen-presenting cells and induces robust CD4+ T-cell responses, particularly in the Th1 phenotype, characterized by cytokine production such as interferon-γ (IFN-γ) and interleukin-2 (IL-2), which are essential for antiviral immunity and long-term immune memory [19,20,21].

RZV is highly effective and safe in both immunocompetent and immunocompromised populations [22], including patients with haematological malignancies [5,23,24,25,26,27] according to clinical trials. Additional studies in patients with cancer and other immunosuppressed settings have confirmed its immunogenicity, supporting its use beyond classical populations such as transplant recipients [26,27,28].

However, immune responses to vaccination in patients with MPNs, particularly those receiving JAK inhibitors, may differ from those observed in other populations. Recent evidence suggests that, despite preserved humoral responses, cellular immune responses to RZV may be attenuated in this setting [29]. This potential dissociation between humoral and cellular immunity remains insufficiently characterized and may have relevant implications for vaccine-induced protection in these patients.

In this context, the present study was designed as a pilot translational analysis to evaluate the immune response to RZV vaccination in patients with MPNs. Specifically, we aimed to assess VZV-specific IgG antibody levels, ex vivo lymphocyte activation profiles and Th1-related cytokine production (IL-2, IL-10 and IFN-γ) before and after vaccination, with particular emphasis on the relationship between humoral and cellular immune responses in this clinically relevant population.

## 2. Results

### 2.1. Cellular Response to RZV Vaccination

**Figure 1:**
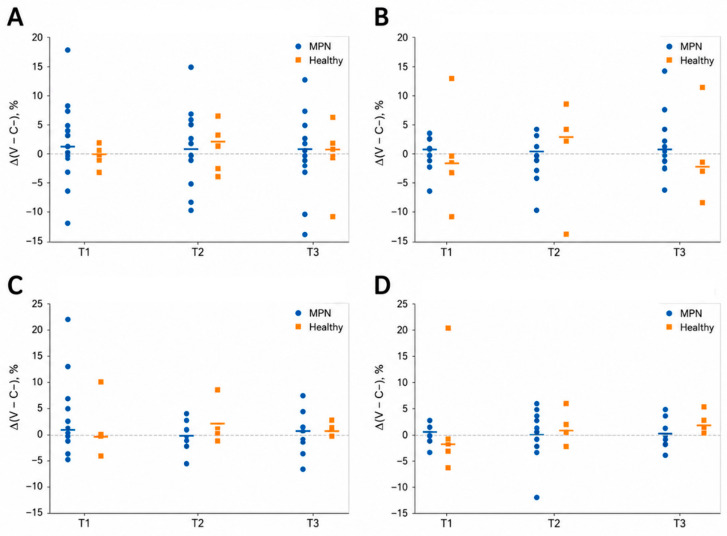
Ex vivo lymphocyte response to RZV antigen stimulation. Antigen-specific cellular response was assessed as the difference in the frequency of each lymphocyte subset between recombinant VZV glycoprotein E-stimulated and unstimulated conditions (Δ[V−C−], percentage points) at three time points: T1 (pre-vaccination), T2 (21 days after the first dose), and T3 (21 days after the second dose). The comparison of cell differentiation frequency was made for the following populations: (**A**) NK cells; (**B**) CD8+ T cells; (**C**) activated CD4+ T cells; and (**D**) CD4+CD25+ T cells. Each dot represents an individual participant. Horizontal lines indicate the median values for each group and time point. Data are shown separately for MPN patients and healthy donors. Selected lymphocyte subsets include CD8+ T cells, activated CD4+ T cells, CD4+CD25+ T cells, and NK cells. Healthy donors are shown for descriptive purposes only. No statistical comparisons were performed. All available data from the 18 MPN patients included in the final cohort are shown. The dashed horizontal line indicates Δ = 0.

Flow cytometric analyses were performed in PBMCs collected at three distinct time points: pre-vaccination (T1), 21 days after the first dose (T2), and 21 days after the second dose (T3), after ex vivo stimulation with recombinant VZV glycoprotein E, to assess changes in the frequency of predefined lymphocyte subsets. Antigen-specific cellular responses were expressed as the difference in the frequency of each lymphocyte subset between recombinant VZV glycoprotein E-stimulated and unstimulated conditions (Δ[V−C−], percentage points). Across MPN patients, responses were heterogeneous and largely driven by individual subjects, without a consistent longitudinal pattern across most lymphocyte subpopulations (Figure 1). Individual longitudinal trajectories for MPN patients are provided in Supplementary Figure S2 to illustrate patient-level kinetics across T1, T2 and T3. In MPN patients, isolated increases in selected lymphocyte subsets were observed in some individuals; however, these changes were inconsistent across patients and time points. Healthy donors showed scattered cellular responses in some subsets, but this observation was purely descriptive and was not intended for formal comparison. These findings should therefore be interpreted as evidence of heterogeneous ex vivo cellular responsiveness rather than as a uniform vaccine-induced cellular activation pattern.

Taken together, these findings indicate a limited and heterogeneous ex vivo cellular response to RZV antigen stimulation in MPN patients, without a consistent pattern of antigen-specific activation.

### 2.2. Cytokine Production

**Figure 2:**
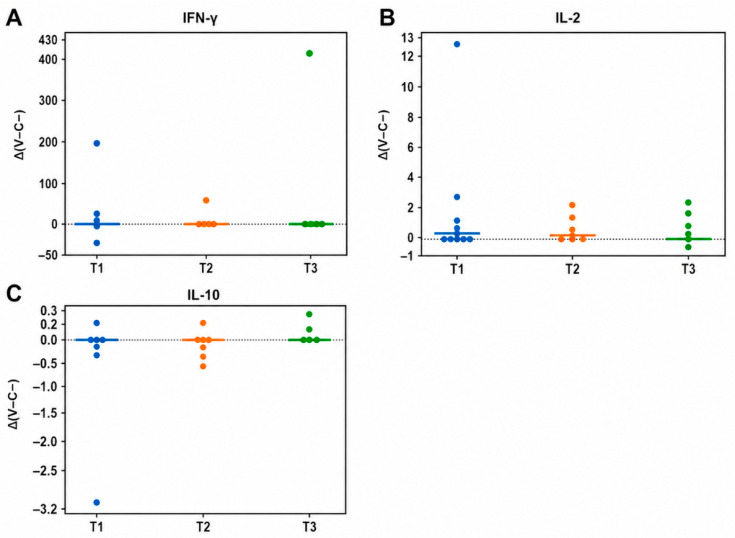
Antigen-specific cytokine response following RZV vaccination. Antigen-specific cytokine production was assessed as Δ(V−C−) in PBMC culture supernatants from the subset of 8 MPN patients with available samples at T1, T2 and T3. Each dot represents an individual patient; all available data are shown. Horizontal lines indicate median values. (**A**) IFN-γ; (**B**) IL-2; and (**C**) IL-10. No statistical comparisons were performed.

Cytokine production was evaluated in a subset of 8 MPN patients for whom sufficient stored PBMC culture supernatant was available after ex vivo stimulation across the evaluated time points. Cytokine responses were therefore considered exploratory and were expressed as antigen-specific response (Δ[V−C−]) at T1, T2 and T3. Overall, IFN-γ, IL-2 and IL-10 responses were low and highly variable across individuals, with no consistent increase over time (Figure 2). IFN-γ production was detectable in a limited number of patients, with marked inter-individual variability and occasional outliers, whereas IL-2 and IL-10 responses remained generally low and close to baseline levels. No consistent temporal pattern or coordinated cytokine response was observed across the three time points. In addition, no clear association was found between cytokine production and the phenotypic lymphocyte changes described above. ConA-induced cytokine production was used as an internal positive-control readout to confirm cellular viability and responsiveness to non-specific bulk T-cell activation and is shown separately in Supplementary Figure S3. Taken together, these findings indicate the absence of a robust and sustained antigen-specific cytokine response following RZV vaccination in MPN patients, supporting the limited cellular activation observed in the ex vivo immunophenotypic analysis.

### 2.3. Humoral Response

**Figure 3:**
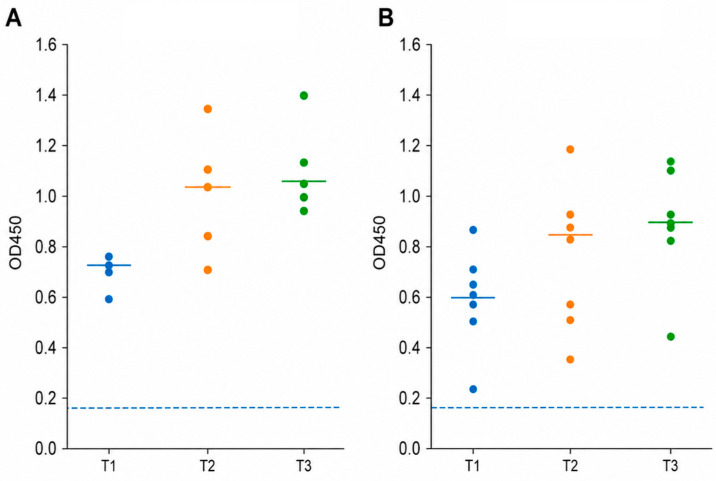
VZV-specific IgG antibody levels after RZV vaccination. VZV-specific IgG antibody levels were measured by ELISA in healthy donors and MPN patients at T1, T2 and T3. Each dot represents an individual subject; horizontal lines indicate the median values. The dashed line indicates the assay cut-off for positivity (OD = 0.162). (**A**) Healthy donors; (**B**) MPN patients. Healthy donors are shown for descriptive purposes only. No statistical comparisons were performed. The dashed horizontal line indicates the assay positivity threshold. Panels (**A**,**B**) are displayed using the same y-axis scale to facilitate visual comparison.

VZV-specific IgG antibody levels were detectable in almost all participants at baseline and increased over time following vaccination in MPN patients, with a similar descriptive trend observed in healthy donors (Figure 3). In healthy donors, antibody levels rose after the first dose and remained high after the second dose, without a clear additional increase. In MPN patients, IgG levels showed an overall upward trend from T1 to T3, although with marked inter-individual variability. At baseline/pre-vaccination (T1), all MPN patients were above the assay positivity threshold for anti-VZV IgG (18/18, 100%). All healthy donors remained above the assay cut-off at the three evaluated time points. In the MPN cohort, nearly all samples remained above the positivity threshold throughout follow-up, with only one patient showing a value below the cut-off at T2 and recovering seropositivity at T3.

Overall, these findings indicate a preserved humoral response pattern following RZV vaccination in MPN patients, with healthy donors showing a similar descriptive trend. Collectively, these findings suggest a discordant pattern between humoral and cellular immune readouts following vaccination in MPN patients.

Exploratory Spearman correlation analyses between IgG levels and cellular responses were performed at each time point in paired MPN samples. No consistent association was observed between humoral and cellular readouts across time points. An isolated positive correlation between IgG levels and CD8+ T-cell responses was observed at T1, but this pattern was not maintained at T2 or T3 (Supplementary Table S1).

## 3. Discussion

The increased incidence of herpes zoster (HZ) in patients with myeloproliferative neoplasms (MPN), particularly those receiving JAK inhibitors, supports the need for effective preventive strategies in this population [7,12]. Despite the availability of the recombinant zoster vaccine (RZV), evidence specifically addressing its immunogenicity in MPN patients remains limited. In this context, the present pilot study provides a focused analysis of both humoral and cellular responses to RZV in MPN patients under real-world treatment conditions.

The main finding of this study is the apparent dissociation between humoral and cellular immune responses following RZV vaccination in MPN patients under JAK inhibitor therapy. Specifically, VZV-specific IgG levels increased over time, whereas antigen-specific cellular responses remained limited, heterogeneous, and not consistently enhanced after vaccination. This pattern suggests that RZV induces a measurable antibody response in MPN patients, whereas the corresponding cellular activation appears to be impaired or highly variable. These findings should be interpreted as indicative of a differential immune pattern under the specific experimental conditions used, rather than definitive evidence of functional dissociation in vivo. This apparent dissociation should be interpreted with caution, as humoral and cellular responses were assessed under different biological contexts: in vivo antibody production versus ex vivo cellular responsiveness after antigenic stimulation. Therefore, these readouts should be considered complementary rather than directly interchangeable. This interpretation is further supported by exploratory correlation analyses, which did not demonstrate a consistent relationship between humoral and cellular immune readouts across time points.

This observation is consistent with the known immunomodulatory effects of JAK inhibitors. Ruxolitinib, the most frequently used treatment in our cohort, has been shown to affect multiple components of the immune system, including T cells and NK cells, through inhibition of the JAK–STAT pathway [11,12,30]. Previous studies have demonstrated that JAK1/2 inhibition can impair T-cell function and cytokine signalling, leading to reduced immune competence in patients with MPN [16,31,32]. In line with these findings, our data show limited activation of CD8+ T cells, NK cells and CD4+CD25+ T-cell subset, together with low and inconsistent cytokine production following antigen stimulation.

Importantly, cytokine analysis further supports the lack of a robust functional response. IFN-γ, IL-2 and IL-10 levels remained low and highly variable across patients, with no consistent temporal increase after vaccination. IFN-γ responses were detectable only in a subset of individuals and were largely driven by isolated cases, whereas IL-2 and IL-10 levels remained close to baseline levels. These findings reinforce the concept that cellular immune activation is not uniformly achieved despite vaccination in this setting. These findings should be interpreted as an ex vivo functional readout under standardized stimulation conditions. This experimental design was intended to provide a functional readout under standardized conditions, although it cannot fully recapitulate the complexity of vaccine-induced immune responses in vivo. Some readouts were influenced by isolated high-response cases, further emphasizing the marked inter-individual variability of the cohort and the exploratory nature of these findings.

The observed immune profile is in agreement with recent reports in patients with haematological malignancies and under targeted therapies. Notably, a recent study in MPN patients treated with JAK inhibitors reported preserved humoral responses but reduced cellular immune responses following RZV vaccination [29]. Although currently available as an abstract, this report provides converging evidence supporting the pattern observed in our cohort. Similar findings have been described in other immunocompromised populations, where RZV induces antibody production, but cellular responses are attenuated or heterogeneous [28,33,34]. These observations should also be interpreted in the broader context of treatment-related immune modulation in MPN, as other therapeutic approaches such as IFN-α have been associated with distinct immunological dynamics [35].

Despite this apparent impairment at the cellular level, the humoral response observed in our study was preserved. Most MPN patients showed IgG levels above the positivity threshold at all time points, with an overall increase after vaccination. However, antibody levels were generally lower than those observed in healthy donors and exhibited greater inter-individual variability. These findings are consistent with previous studies in haematological malignancies, where vaccination induces seroconversion in a high proportion of patients but with reduced magnitude compared to controls [25,33,34].

However, the serological findings should be interpreted cautiously because the ELISA used in this study measured total anti-VZV IgG and did not specifically quantify antibodies against glycoprotein E, the main antigenic component of RZV. Therefore, baseline seropositivity and post-vaccination increases may partly reflect boosting of pre-existing VZV immunity rather than exclusively vaccine-specific responses.

Taken together, our findings suggest that RZV vaccination in MPN patients under JAK inhibitor therapy is associated with a detectable humoral response, whereas the cellular readouts assessed in this study did not show a consistent pattern of antigen-specific activation. This dissociation may have clinical implications, as protection against HZ is thought to rely not only on antibody production but also on effective cell-mediated immunity.

This observation raises the possibility that, despite achieving seropositivity, the immune response captured by the analytical approaches used in this study may remain incomplete at the cellular level. Although clinical protection cannot be inferred from immunological data alone, the limited and heterogeneous cellular responses observed in this study suggest that antibody levels may not fully reflect functional immunity in this setting.

This study has several limitations inherent to its pilot design. The sample size was small, particularly for cytokine analyses, and the cohort was heterogeneous in terms of disease subtype and treatment exposure. Cellular responses were assessed using an ex vivo stimulation approach and a focused flow cytometry panel, which may not fully capture the complexity of vaccine-induced immunity in vivo. In addition, NK cells were analysed as a total CD3−CD56+ population, and CD56^bright^/CD56^dim^ subsets were not evaluated because the panel and event numbers were not optimized for this purpose. Finally, the ELISA used in this study measured total anti-VZV IgG rather than gE-specific antibodies, limiting the ability to attribute humoral changes exclusively to RZV-specific responses.

Despite these limitations, these findings raise concerns about relying solely on serological markers and provide a coherent and biologically plausible framework for understanding immune responses to RZV in MPN patients. The consistent pattern observed across phenotypic, functional and humoral analyses supports the hypothesis that JAK inhibition differentially affects immune compartments, preserving antibody production while impairing cellular immune activation.

Further studies incorporating antigen-specific assays and longitudinal clinical outcomes, in larger, well-characterized cohorts, will be required to determine the clinical relevance of this immune dissociation and to optimize vaccination strategies in this high-risk population.

## 4. Materials and Methods

### 4.1. Patients

In this prospective pilot study, 18 patients diagnosed with MPN, including myelofibrosis (MF), polycythaemia vera (PV) and essential thrombocythemia (ET), and an age-matched small group of healthy donors (*n* = 4) were included. The healthy donor group included two males and two females, with a mean age of 68.0 ± 7.7 years. Healthy donors were included as a descriptive reference group only and were not considered for formal comparative analyses because of the limited sample size. Patients were recruited from routine clinical practice. The inclusion criteria comprised a confirmed diagnosis of MPN according to the WHO criteria and eligibility for vaccination. Most patients were receiving ruxolitinib at the time of inclusion (15/18, 83.3%). The remaining three patients were receiving other therapies, including momelotinib, interferon-based therapy and prednisone. Due to the limited number of patients not receiving ruxolitinib, subgroup analyses according to treatment exposure were not performed. Table 1 summarizes the baseline demographic and clinical characteristics of the MPN cohort.

### 4.2. Ethical Statement

The study was reviewed and approved by the Comité de Ética de la Investigación Provincial de Málaga, which evaluated the proposal entitled “Estudio observacional prospectivo: inmunogenicidad y eficacia de las vacunas frente al herpes Zoster en pacientes onco-hematológicos tratados con ANTIJAK” in its session held on 28 September 2023. The study was conducted in accordance with the Declaration of Helsinki. Blood samples were obtained during routine clinical care and processed under standardized biobanking conditions at the ECAI Biobanco Provincial de Málaga. All sample collection, handling, and storage followed institutional standard operating procedures (SOPs) to ensure sample quality and assay reproducibility. All patients received detailed written information about the study and provided signed informed consent before participation, in accordance with institutional and international ethical guidelines.

### 4.3. Vaccination and Blood Sample Collection

**Figure 4:**
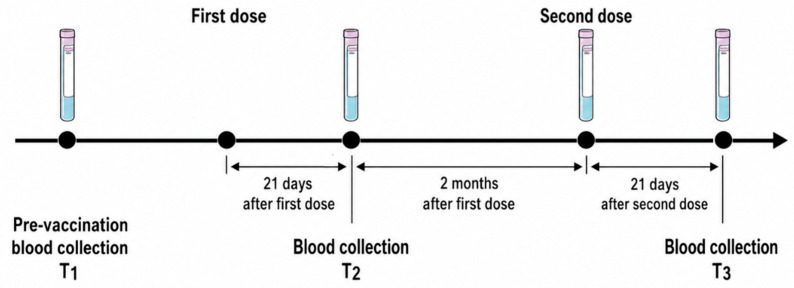
Vaccination and blood collection timeline. Peripheral blood samples from patients with MPN were collected at three time points: T1, before vaccination; T2, 21 days after the first RZV dose; and T3, 21 days after the second RZV dose. The second dose was administered approximately two months after the first dose, and T3 was collected approximately 60 days after T2.

Participants received two doses of recombinant zoster vaccine (RZV; Shingrix^®^, GlaxoSmithKline, London, UK) administered intramuscularly, with the second dose administered approximately two months after the first dose. Two EDTA tubes of peripheral blood were collected at three time points: T1, before vaccination; T2, 21 days after the first dose; and T3, 21 days after the second dose, corresponding to approximately 60 days after T2 (Figure 4). Blood was collected in EDTA tubes and processed on the same day.

### 4.4. Isolation of Peripheral Blood Mononuclear Cells (PBMCs)

Peripheral blood was diluted 1:1 with physiological saline and PBMCs were isolated using Histopaque-1077 (Sigma-Aldrich, St. Louis, MO, USA) density gradient centrifugation (400× *g*, 30 min, room temperature). Plasma was collected and stored at −80 °C for subsequent serological analysis. PBMCs were washed, resuspended in RPMI 1640 medium supplemented with 10% heat-inactivated fetal bovine serum and 2 mM L-glutamine, and counted using trypan blue exclusion.

### 4.5. PBMC Culture and Ex Vivo Stimulation

PBMCs (3 × 10^5^ cells/well) were cultured in 96-well plates under three conditions: negative control (medium only, C−); antigen stimulation with recombinant VZV glycoprotein E (5 µg/mL, V); and positive control with ConA (5 µg/mL; used as an internal quality control to verify cell viability and functional responsiveness after the 5-day culture period). Cells were incubated for 5 days at 37 °C and 5% CO_2_. This ex vivo stimulation approach was used to assess the functional responsiveness of immune cells to antigenic challenge, rather than to directly reflect in vivo immune activity. This strategy was selected to increase the likelihood of detecting low-frequency antigen-responsive cellular signals in a pilot setting. After incubation, supernatants were collected and stored at −80 °C for cytokine analysis, and cells were processed for flow cytometry.

### 4.6. Flow Cytometry

After the 5-day culture period, recovered cells from each stimulation condition were stained with fluorochrome-conjugated antibodies against CD45, CD3, CD4, CD8, CD19, CD56, CD69 and CD25 (BD Biosciences, San Jose, CA, USA) and analysed using a CytoFLEX flow cytometer (Beckman Coulter, Hong Kong, China). PBMCs had been plated at 3 × 10^5^ cells/well before stimulation, and a total of 20,000 events per sample were acquired. First, lymphocytes were selected according to forward- and side-scatter properties, followed by singlet discrimination using FSC-A versus FSC-H and identification of CD45+ leukocytes. From this CD45+ population, major lymphocyte lineages were defined as follows: T cells were identified as CD45+CD3+CD19− cells, B cells as CD45+CD3−CD19+ cells, and NK cells as CD45+CD3−CD56+ cells. CD4+ and CD8+ T-cell subsets were identified within the CD3+ T-cell population. CD69 was used to identify activated CD4+ and CD8+ T-cell subsets, whereas CD25 was used to identify the CD4+CD25+ T-cell subset. Selected lymphocyte subsets were defined according to the marker combinations shown in Table 2. Antigen-specific cellular response was quantified as the difference in cellular frequency between recombinant VZV glycoprotein E-stimulated and unstimulated conditions (Δ[V−C−], percentage points) for each lymphocyte subset and time point. Δ(V−C−) was calculated from proportions/frequencies and not from absolute cell numbers or MFI values. A schematic gating workflow is provided in Supplementary Figure S1. NK cells were analysed as a total CD45+CD3−CD56+ population. Subdivision into CD56^bright^/CD56^dim^ NK-cell subsets was not performed because this was not part of the predefined analysis strategy, CD16 was not included in the antibody panel, and event numbers were insufficient in some samples for reliable NK-cell subset stratification.

### 4.7. IgG Antibody Measurement

Plasma VZV-specific IgG levels were measured using a commercial ELISA kit (Elabscience, Wuhan, China). Samples were diluted 1:10 and processed according to the manufacturer’s instructions. Optical density (OD) was measured at 450 nm. Results were expressed as individual OD values and compared across time points (T1, T2, T3). The positivity cut-off was defined according to manufacturer specifications.

### 4.8. Cytokine Quantification

Cytokine levels (IFN-γ, IL-2 and IL-10) were measured in PBMC culture supernatants using a Human Magnetic Luminex Performance Assay Base Kit A (R&D Systems, Minneapolis, MN, USA, LUHM000), with analyte-specific components for IFN-γ (LUHM285), IL-2 (LUHM202), and IL-10 (LUHM217). The antigen-specific cytokine response was calculated as the difference between recombinant VZV glycoprotein E-stimulated and unstimulated conditions (Δ[V−C−]) for each cytokine and time point.

### 4.9. Statistical Analysis

Given this pilot study’s exploratory nature and limited sample size, no formal inferential statistical analyses were performed. Data are presented to illustrate distribution, variability and individual response patterns across time points. This approach was deliberately chosen to avoid overinterpretation of underpowered comparisons. Exploratory correlations between humoral (IgG levels) and cellular responses (Δ[V−C−]) were assessed using Spearman rank correlation at each time point in paired MPN samples. All analyses and graphical representations were performed using GraphPad Prism v10.0 (GraphPad Software, Boston, MA, USA). Outlying values were retained in all analyses due to the exploratory design and limited sample size of the study.

## Figures and Tables

**Table 1:** Baseline characteristics of the study population (*n* = 18).

Variable	Category	*n* (%)
Sex	Male	10 (55.6%)
	Female	8 (44.4%)
Diagnosis	MF	8 (44.4%)
	PV	9 (50.0%)
	ET	1 (5.6%)
Treatment	Ruxolitinib	15 (83.3%)
	Other	3 (16.7%)
Age (years)	Mean ± SD	68.3 ± 9.2

**Table 2:** Lymphocyte classification into major populations based on marker combinations.

Surface Markers	Population	Abbreviation
CD45+CD3+CD19−	T Lymphocytes	T cells
CD3+CD4+	T-helper or CD4+ cells	CD4+
CD3+CD4+CD69+	Activated CD4+ T cells	CD4+ act
CD3+CD8+	Cytotoxic T lymphocytes or CD8+ cells	CD8+
CD3+CD8+CD69+	Activated CD8+ T cells	CD8+ act
CD3+CD4+CD25+	CD4+CD25+ T-cell subset	CD4+CD25+
CD45+CD3−CD19+	B lymphocytes	B cells
CD45+CD3−CD56+	Natural killer cells	NK

## Data Availability

The original contributions presented in this study are included in the article/Supplementary Material. Further inquiries can be directed to the corresponding author.

## Supplementary Materials

The following supporting information can be downloaded at: https://www.mdpi.com/article/10.3390/ijms27125543/s1.

## Abbreviations

The following abbreviations are used in this manuscript:

Abbreviation	Meaning
ConA	Concanavalin A
ELISA	Enzyme-linked immunosorbent assay
ET	Essential thrombocythemia
gE	Glycoprotein E
HZ	Herpes zoster
IFN-γ	Interferon-gamma
IgG	Immunoglobulin G
IL-2	Interleukin-2
IL-10	Interleukin-10
JAK	Janus kinase
JAKi	Janus kinase inhibitor
JAK/STAT	Janus kinase/signal transducer and activator of transcription
MF	Myelofibrosis
MPN	Myeloproliferative neoplasms
NK	Natural killer
OD	Optical density
PBMCs	Peripheral blood mononuclear cells
PV	Polycythaemia vera
RZV	Recombinant zoster vaccine
V	Antigen-stimulated condition
VZV	Varicella-zoster virus
WHO	World Health Organization
