# Supplementary material for: Dissociated Humoral and Cellular Immune Responses to Recombinant Zoster Vaccine in Myeloproliferative Neoplasms Under JAK Inhibition: A Pilot Study
Source: Int J Mol Sci. 2026 Jun 19;27(12):5543. doi: 10.3390/ijms27125543 (PMC13300440; doi:10.3390/ijms27125543)
Supplement: Supplementary file 1 [file ijms-27-05543-s001.zip › ijms-4335853-supplementary.pdf]

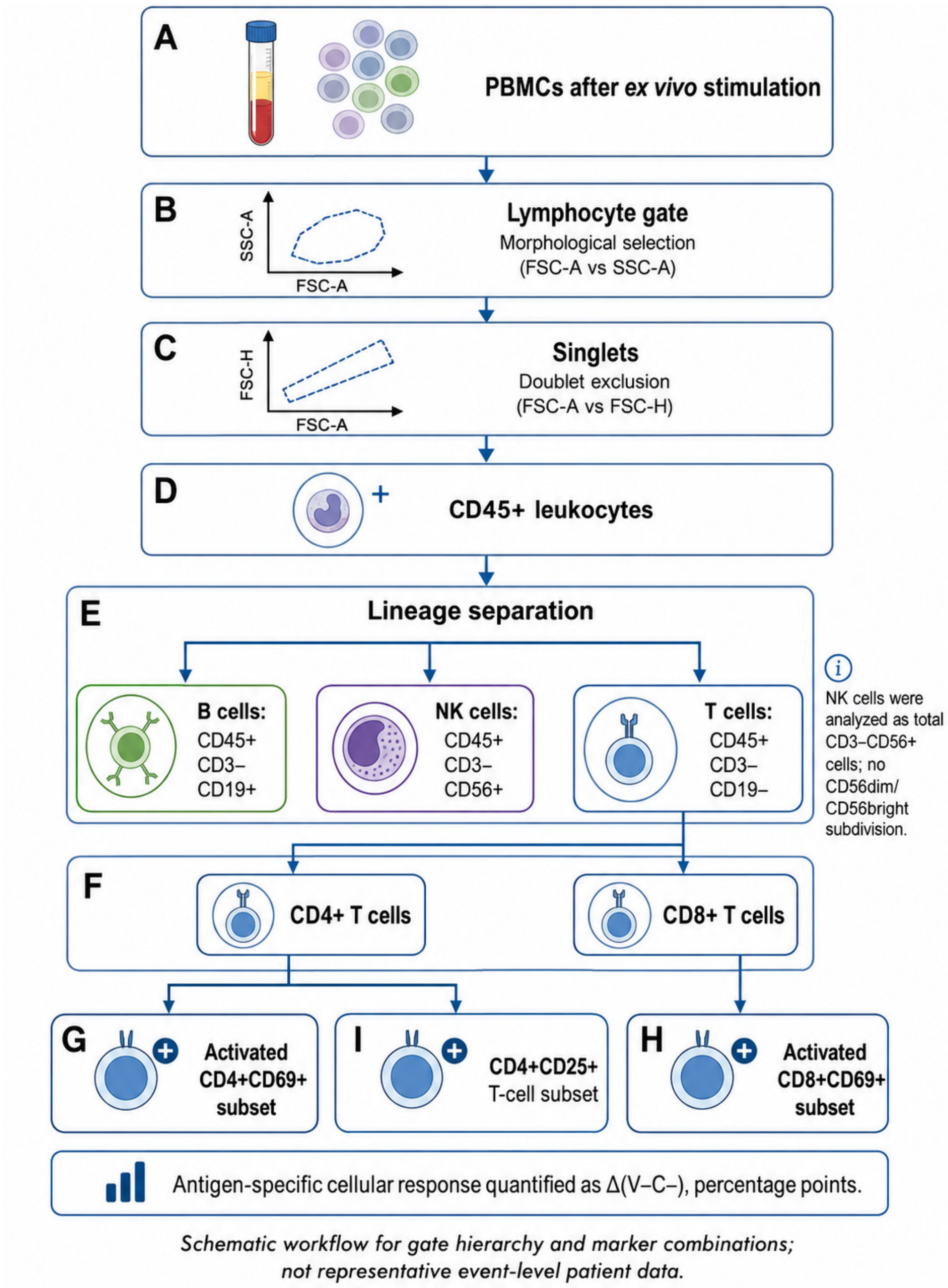

Figure S1. Schematic flow cytometry gating workflow for lymphocyte subset identification.

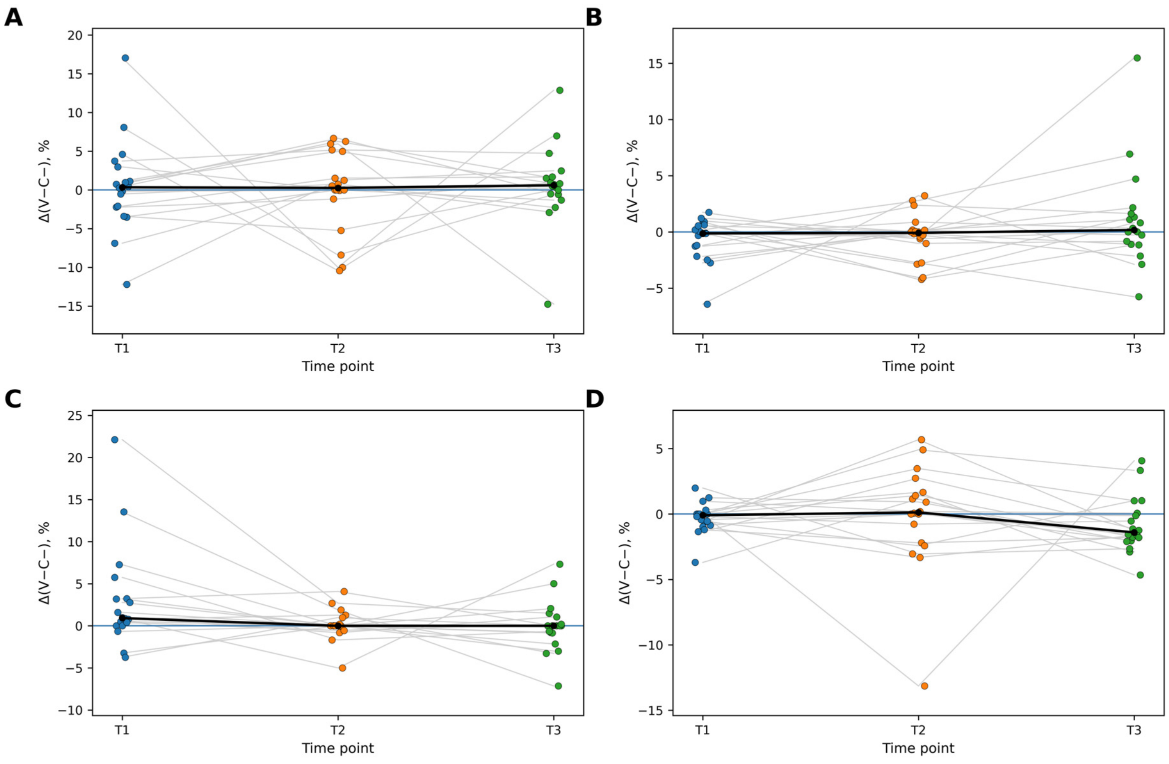

Figure S2. Individual longitudinal trajectories of antigen-specific cellular responses in MPN patients.

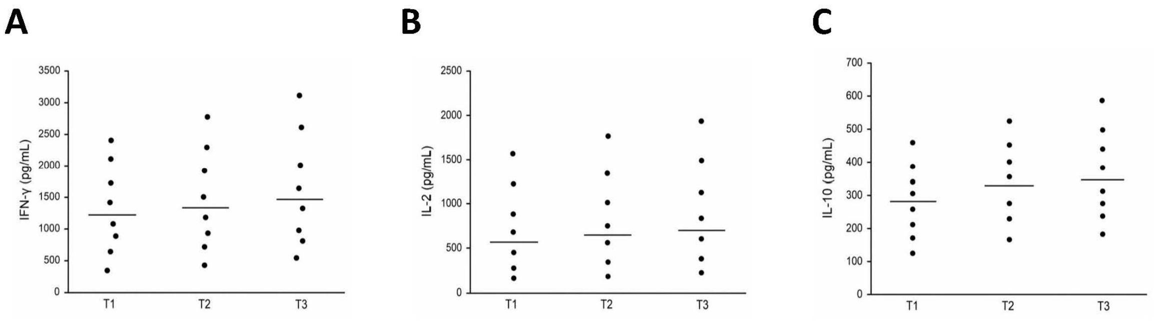

Figure S3. ConA-induced cytokine responses used as positive-control readout for PBMC culture responsiveness.

Table S1. Exploratory Spearman correlations between IgG levels and antigen-specific cellular responses in MPN patients by time point.

Time	Cellular_readout	n_pairs	Spearman_rho	p_value
**T1**	NK	18	-0.349	0.143
**T2**	NK	18	-0.232	0.339
**T3**	NK	18	-0.093	0.705
**T1**	CD8+ T cells	18	0.535	0.018
**T2**	CD8+ T cells	18	0.088	0.721
**T3**	CD8+ T cells	18	-0.244	0.314
**T1**	Activated CD4+ T cells	18	0.256	0.29
**T2**	Activated CD4+ T cells	18	-0.237	0.328
**T3**	Activated CD4+ T cells	18	-0.354	0.137
**T1**	CD4+CD25+ T cells	18	0.012	0.96
**T2**	CD4+CD25+ T cells	18	-0.023	0.926
**T3**	CD4+CD25+ T cells	18	0.2	0.412
